# Dying endothelial cells stimulate proliferation of malignant glioma cells via a caspase 3-mediated pathway

**DOI:** 10.3892/ol.2013.1223

**Published:** 2013-03-01

**Authors:** PING MAO, LUKE SMITH, WANFU XIE, MAODE WANG

**Affiliations:** 1 Department of Neurosurgery, The First Affiliated Hospital, School of Medicine, Xi’an Jiaotong University, Xi’an, Shaanxi 710061, P.R. China;; 2 Department of Neurological Surgery, The Ohio State University Medical Center, Columbus, OH 43210, USA

**Keywords:** glioma, endothelial cells, cell proliferation, caspase 3

## Abstract

Emerging evidence has indicated that apoptotic cells have a compensatory effect on the proliferation of neighboring cells. However, the potential role of dying vascular endothelial cells (ECs) in glioma tumor proliferation remains unclear. In the present study, three glioma cell lines were cocultured with dying ECs under various conditions to evaluate the effect of dying ECs on tumor proliferation using alamarBlue and trypan blue assays to assess cell proliferation and viability, respectively. The results suggested that dying ECs had a marked ability to facilitate glioma cell growth via a caspase 3-mediated pathway. Furthermore, calcium-independent phospholipase A_2_ (iPLA_2_), a downstream gene regulated by caspase 3, is highly involved in this process. Prostaglandin E_2_ (PGE_2_) was the final effector of the caspase 3-iPLA_2_ signaling pathway in glioma cell proliferation. Knockdown of caspase 3 or iPLA_2_ using shRNA negated the growth stimulating effect of dying ECs. By contrast, the overexpression of iPLA_2_ in ECs via the pLEX lentiviral vector system or addition of PGE_2_ into culture medium had a growth promoting effect on glioma cells. Overall, the present data revealed a paracrine signal released from dying ECs which promotes the proliferation of surrounding glioma cells, demonstrating the importance of blocking compensatory proliferation during tumor therapy. Additionally, targeting caspase 3-mediated pathways combined with current therapeutic strategies may be a promising approach for improving the dismal prognosis associated with these malignant tumors.

## Introduction

Decades of studies have indicated that the tumor microvasculature has an essential role in malignant tumor growth and progression (1–3). Thus, multiple anti-angiogenic compounds have been designed to increase the therapeutic efficacy of tumor treatments. However, this anti-angiogenic strategy has not significantly improved the prognosis of patients suffering from malignant glioma, according to clinical results (4) which indicated that damaged vascular endothelial cells (ECs) may have an unidentified role in tumor maintenance or even proliferation.

Researchers have made a concerted effort to understand how the angiogenic process is regulated by tumor cells under various conditions (5,6). However, a limited number of studies have focused on the potential effect of dying ECs on tumor cell biology. One study reported that the presence of damaged ECs regulated tumor response to radiotherapy by facilitating *in vivo* tumor growth and contributing to therapy resistance (7). However, the mechanistic details remain unclear. Another group observed a proliferative effect of apoptotic cells on wound healing processes, and the caspase 3-mediated ‘phoenix rising’ pathway was found to be involved in this compensatory proliferation (8).

We hypothesized that ECs exposed to lethal factors had an enhanced ability to support the proliferation of glioma cells. Furthermore, the caspase family, which is highly involved in cell apoptosis, may be the key regulator of this process. To investigate our hypothesis, the effects of ECs on the proliferation of glioma cells were evaluated under various conditions. Based on the present data, it was demonstrated that dying ECs were able to accelerate glioma cell growth via a caspase 3-mediated pathway which presented a novel insight into the interaction between damaged vascular ECs and surrounding tumor cells.

## Materials and methods

### Cell culture

Three glioma cell lines (U87MG, U251MG and C6MG) and human umbilical vascular ECs (HUVECs) were used in the present study. All these cells were purchased from the American Type Culture Collection (ATCC; Manassas, VA, USA). Cells were cultured in Dulbecco’s modified Eagle’s medium (DMEM; Gibco-Invitrogen, Carlsbad, CA, USA) supplemented with 10% fetal bovine serum (FBS; Gibco-Invitrogen) under normoxic conditions. In coculture conditions, hypoxia-exposed or untreated HUVECs were seeded on 24-well inserts (0.4 *μ*m; Millipore, Billerica, MA, USA) and glioma cells were planted on a 24-well receiver plates (Millipore). For hypoxia exposure, HUVECs were cultured under several hypoxic conditions, including 6, 2 and 1% O_2_ using a hypoxia incubator (Heracell 150; Thermo Scientific, Waltham, MA, USA). The conditional medium was harvested from HUVECs exposed to 2% O_2_ for 48 h and the glioma cell lines were then cultured in the conditional medium for 8 days before the fluorescence intensity was measured. This study was approved by the ethics committee of Xi’an Jiaotong University Medical School, China.

### Cell proliferation assay

AlamarBlue assays were performed to evaluate the proliferation of glioma cells according to the manufacturer’s instructions (Invitrogen, Carlsbad, CA, USA). Glioma cells were seeded on 24-transwell receiver plates at a density of 1.0×10^3^ cells/well. For the coculture system, ∼1.0×10^5^ pretreated HUVECs were seeded on each companion insert. The fluorescence intensity of the glioma cells was measured with a microplate reader (FLUOstar OPTIMA, BMG Labtech, Offenburg, Germany) after an 8-h incubation with 10% alamarBlue reagent added into each well.

### Cell viability assay

Trypan blue exclusion tests were performed to assess the cell viability following exposure to hypoxic conditions. HUVECs were cultured on 24-well plates under various hypoxic conditions (6, 2 and 1% O_2_) at a density of 1.0×10^5^ cells/well with the addition of 10% trypan blue reagent (Invitrogen). The HUVEC viability was then quantified by counting the viable and dead cells using a hemocytometer at various time points (12, 24, 36 and 48 h).

### Gene transduction

Lentivirus vectors encoding shRNA against caspase 3, caspase 6 and calcium-independent phospholipase A_2_ (iPLA_2_) were used for gene knockdown according to the manufacturer’s instructions (Sigma, St. Louis, MO, USA). The following sequences were used in this study: shCASP3 #1, CCGGGTGGAATTGATGCGTGATGTTCTCG AGAACATCACGCATCAATTCCACTTTTT; shCASP3 #2, CCGGGCGAATCAATGGACTCTGGAACTCGAGTTCCA GAGTCCATTGATTCGCTTTTT; shCASP6 #1, CCGGGT TAGGGTGAAGCATTATGGTCCGAGACCATAATGCTTC ACCCTAACTTTTT; shCASP6 #2, CCGGGCTTTGTG TGTGTCTTCCTGACTCGAGTCAGGAAGACACACACA AAGCTTTTT; shPLA2G6, CCGGCCTACTTACTTC CGACCCAATCTCGAGATTGGGTCGGAAGTAAGTAGG TTTTTG. Additionally, the pLEX lentiviral vector system (Open Biosystems, Waltham, MA, USA) was used to deliver activated (ac) iPLA_2_ into ECs according to manufacturer’s instructions. A truncated version of mouse iPLA_2_ was amplified with RT-PCR using the following primers: forward, GACTAGTGCCACCATGCAGCACCAAGGACCTCTTC GACTG; reverse, ATAAGAATGCGGCCGCGTCCACGA CCATCTTGCCCAG. Pfx polymerase (Invitrogen) was used for the PCR amplification. The amplified fragment encoded aa453–679 of murine iPLA_2_ (equivalent to aa514–733 of human iPLA_2_) which has been demonstrated to be a constitutively active caspase cleavage product (9,10). In all cases, 293T cells were used to produce viable and recombinant lentiviral vectors according to manufacturer’s instructions.

### Western blot analysis

Immunoblotting was performed to analyze sample lysates containing protease inhibitor cocktail (Sigma). The BCA Protein Assay kit (Pierce-Thermo Scientific, Rockford, IL, USA) was used to measure the protein concentrations and equal amounts of proteins were loaded onto SDS-PAGE gels (NuPAGE-Invitrogen) for the electrophoresis and transfer procedures. PVDF membranes (Invitrogen) were then incubated overnight in a cold room with multiple primary antibodies, including caspase 3 (Rabbit, 1:1,000; Abcam, Cambridge, UK), caspase 6 (Rabbit, 1:1,000; Abcam), iPLA_2_ (Rabbit, 1:500; Abcam) and β-actin (Rabbit, 1:1,000; Abcam). Signals were amplified using anti-rabbit secondary antibody (Goat, horseradish peroxidase-conjugated, 1:2,000–1:4,000; Abcam) followed by detection of enhanced chemiluminiscence.

### ELISA

The prostaglandin E_2_ (PGE_2_) production of HUVECs under various conditions was evaluated using a PGE_2_ ELISA kit (Abcam). Pretreated or untreated HUVECs were seeded on 6-well plates at a density of 1.0×10^5^ cells/well. Cells were cultured with DMEM supplemented with 10% FBS. After a 24-h incubation, the PGE_2_ concentrations in the supernatants were measured according to the manufacturer’s instructions.

### Statistical analysis

Student’s t-tests and one-way ANOVA were performed to analyze the data using SPSS 17.0 software (IBM, Armonk, NY, USA). P<0.05 was considered to indicate statistically significant differences.

## Results

### Growth stimulating effect of dying ECs on glioma cells

**Figure 1:**
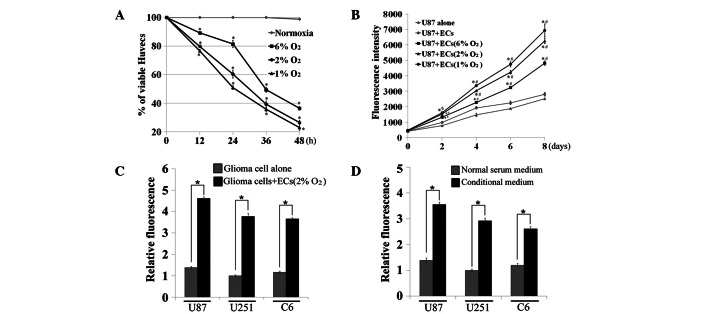
Growth stimulating effect of dying ECs on glioma cells. (A) HUVEC viability was significantly reduced following various hypoxic exposures, particularly at 48 h (^*^P<0.05 vs. normoxic conditions, between 12 and 48 h). Cell viability was evaluated by counting viable and dead cells using a hemocytometer following the addition of 10% trypan blue. (B) Growth of U87MG cells was significantly promoted by coculture with dying ECs (^*^P<0.05 vs. U87MG alone group; ^#^P<0.05 vs. U87MG and EC coculture group; between days 2 and 8). Hypoxic exposure was used to induce cell death and apoptosis (2% O_2_, 48 h). Cell growth was evaluated by fluorescence intensity. (C) Growth stimulating ability of dying ECs on three glioma cell lines. ECs were pretreated with 2% O_2_ exposure for 48 h (^*^P<0.05). (D) Growth stimulating effect of conditional medium on three glioma cell lines. Conditional medium was harvested from dying ECs cultured under 2% O_2_ conditions for 48 h (^*^P<0.05). All error bars represent SEM, n=4. EC, endothelial cell; HUVEC, human umbilical vascular endothelial cell.

To examine our hypothesis, a series of experiments were performed to investigate the effects of dying vascular ECs on the proliferation of malignant glioma cells. First, there was a significant decrease in EC viability following exposure to various severe hypoxic conditions (6, 2 and 1% O_2_) at various times, particularly 48 h (P<0.05, one-way ANOVA, Fig. 1A). U87MG cells were cocultured with pretreated (hypoxia exposure for 48 h) or untreated ECs on 24-transwell plates for up to 8 days, then fluorescence intensity was measured to evaluate U87MG cell proliferation rates. The results showed that dying ECs had a more marked ability to stimulate the growth of cocultured U87MG cells compared with normal ECs or U87MG cells alone (P<0.05 vs. U87MG alone group; P<0.05 vs. U87MG and EC coculture group; one-way ANOVA, between days 2 and 8, Fig. 1B). Furthermore, this effect occurred in a dose-dependent manner whereby ECs pretreated with higher levels of hypoxia showed enhanced growth promoting ability compared with groups with no treatment or lower levels of hypoxic exposure. To support these observations, three glioma cells lines were cocultured with dying ECs (2% O_2_ exposure for 48 h) and the same growth promoting effects as previously described were demonstrated (P<0.05, t-test, Fig. 1C). Next, the conditional medium was harvested from dying ECs and the growth stimulating effect of this on three glioma cell lines was investigated. The addition of the conditional medium increased the glioma cell growth more than two-fold compared with normal serum medium (P<0.05, t-test, Fig. 1D).

### Caspase 3 regulated growth stimulating signal in dying ECs

**Figure 2:**
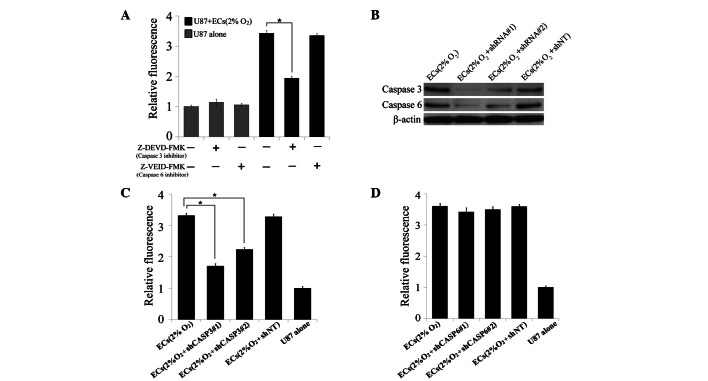
Key role of caspase 3 in regulating the growth stimulating signal released from dying ECs. (A) Blocking effects of selective caspase 3 and caspase 6 inhibitors on the growth stimulating effect of dying ECs on U87MG cells (^*^P<0.05). Z-DEVD-FMK, a caspase 3 inhibitor (100 *μ*M), or Z-VEID-FMK, a caspase 6 inhibitor (100 *μ*M) were added into the 24-well plates. (B) Western blot analysis of caspase 3 and caspase 6 expression levels in dying ECs following various treatments. shRNA without a target (shNT) was used as a negative control. (C) Knockdown of caspase 3 significantly inhibited the growth promoting effect of dying ECs on U87MG cells (^*^P<0.05). (D) Knockdown of caspase 6 showed no effect on the growth stimulating signal from dying ECs. All error bars repesent SEM, n=4. EC, endothelial cell.

Emerging evidence has shown that caspase 3 is the key regulator in compensatory proliferation during the wound healing process (8). Thus we hypothesized that the caspase family may also be involved in the growth stimulating signal released from dying ECs. First, the blocking effect of two specific caspase inhibitors on the growth stimulating signals from dying ECs was examined. Notably, caspase 3 inhibitor (Z-DEVD-FMK, 100 *μ*M), but not caspase 6 inhibitor (Z-VEID-FMK, 100 *μ*M), blocked the growth enhancing effect of dying ECs on U87MG cells (P<0.05, t-test, Fig. 2A). For further confirmation, shRNA against caspase 3 and caspase 6 was transduced into ECs which were then subjected to hypoxic exposure. Western blot analysis was performed to demonstrate the knockdown effect of caspase 3 and caspase 6 in the ECs (Fig. 2B). Knockdown of caspase 3 significantly blocked the growth stimulating signal in ECs following severe hypoxic pretreatment (P<0.05, t-test, Fig. 2C), but no effects were observed in the caspase 6 knockdown group (Fig. 2D). These results indicate that caspase 3 is a key regulator in the growth stimulating process.

### Involvement of iPLA_2_ in caspase 3-mediated growth stimulation of glioma cells

**Figure 3:**
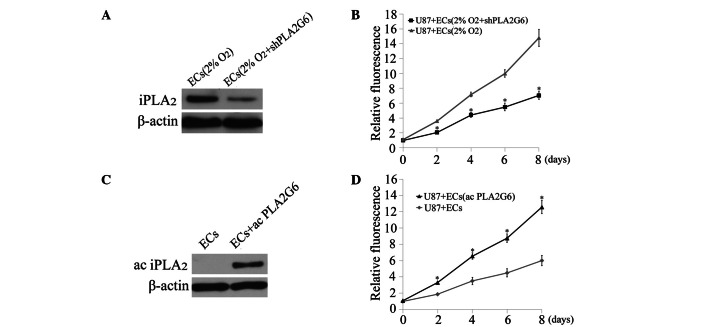
Involvement of iPLA_2_ in caspase 3-mediated growth stimulating pathway in dying ECs. (A) Immunoblot analysis of iPLA_2_ expression in dying ECs following PLA2G6 knockdown. (B) Growth stimulating effect of dying ECs on U87MG cells was reduced by knockdown of PLA2G6 (^*^P<0.05, between days 2 and 8). (C) Expression levels of ac iPLA_2_ in ECs following PLA2G6 overexpression. (D) Overexpression of PLA2G6 in ECs significantly promoted the proliferation of U87MG cells compared with wild type ECs (^*^P<0.05, between days 2 and 8). All error bars represent SEM, n=4. iPLA_2_, calcium-independent phospholipase A_2_; EC, endothelial cell; ac, active.

To further understand the mechanism of the growth stimulating process, group 6 of the iPLA_2_ family encoded by PLA2G6, a downstream target of caspase 3 (10), was investigated. First, shRNA against PLA2G6 was transduced into ECs which were then subjected to hypoxia exposure. Western blot analysis showed decreased levels of the iPLA_2_ signal in dying ECs following PLA2G6 knockdown (Fig. 3A). The effects of dying ECs with or without PLA2G6 knockdown on the proliferation of U87MG cells were then compared. A significant reduction in the growth stimulating effect was observed in the PLA2G6 knockdown group (P<0.05, t-test, Fig. 3B). To further investigate the involvement of iPLA_2_ in the growth stimulating process, an expression vector containing the PLA2G6 gene was transduced into ECs to increase the PLA2G6 expression level (Fig. 3C). ECs overexpressing PLA2G6 exhibited a more marked ability to promote the proliferation of U87MG cells under coculture conditions compared with normal ECs (P<0.05, t-test, between days 2 and 8, Fig. 3D).

### Important role of PGE_2_ in the growth stimulating process

**Figure 4:**
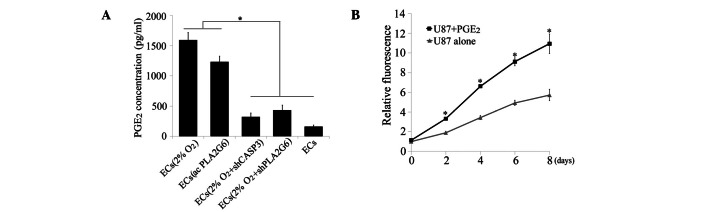
Correlation of PGE_2_ with the caspase 3-iPLA_2_ pathway in the stimulation of U87MG proliferation induced by dying ECs. (A) Level of PGE_2_ in ECs under various conditions (^*^P<0.05). PGE_2_ concentrations were evaluated using an ELISA kit. (B) Effect of adding PGE_2_ into culture medium on the growth promotion of U87MG cells (^*^P<0.05, between days 2 and 8). All error bars represent SEM, n=4. PGE_2_, prostaglandin E_2_; iPLA_2_, calcium-independent phospholipase A_2_; EC, endothelial cell.

As previously described, it was observed that the conditional medium from dying ECs also had the ability to facilitate the growth of glioma cells, indicating the secretion of growth factors during this process. Consequently, the concentration of PGE_2_, a downstream growth factor of iPLA_2_(11), was measured in the supernatants obtained from the various EC groups. Severe hypoxic exposure and overexpression of PGE_2_ in ECs significantly increased the production of PGE_2_ compared with normal ECs (P<0.05, one-way ANOVA, Fig. 4A). By contrast, knockdown of caspase 3 or PLA2G6 blocked the signal that increased PGE_2_ production in dying ECs. Next, U87MG cells were treated with 1,500 pg/ml PGE_2_ added to the culture medium which significantly promoted the growth of U87MG cells compared with no treatment (P<0.05, t-test, between days 2 and 8, Fig. 4B).

**Figure 5:**
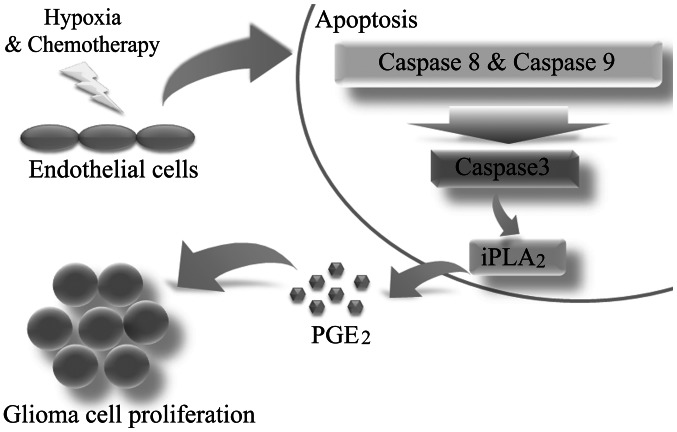
Caspase 3 mediated pathway regulating the growth stimulating signal from dying ECs. iPLA_2_, calcium-independent phospholipase A_2_; PGE_2_, prostaglandin E_2_; EC, endothelial cell.

Overall, the present study has described the growth stimulating ability of dying ECs surrounding malignant glioma cells. Furthermore, caspase 3 was identified as the key regulator of this process (Fig. 5).

## Discussion

Previously, it was considered that apoptosis was an isolated process in apoptotic cells which did not affect neighboring cells (12,13). However, emerging evidence indicates that dying cells are not inactive and have the ability to accelerate the growth of surrounding cells, termed ‘compensatory proliferation’ (14–16). In the present study, the presence of dying ECs was observed to possess a clear growth promoting effect on three glioma cell lines. Furthermore, this process was under the control of caspase 3, an executor during cell apoptosis, but not caspase 6. The gene iPLA_2_, a downstream target of caspase 3, was reported to regulate the release of arachidonic acid which is further modified into eicosanoids such as PGE_2_(17). Additionally, PGE_2_ is known to be an important growth factor and regulator in cell biology (18,19). Taken together, this evidence reveals the key role of the caspase 3-iPLA_2_-PGE_2_ signaling pathway in the growth stimulation process, as demonstrated by *in vitro* gene knockdown and overexpression. Although these results appear contrary to the predominant view of apoptotic cells and caspase 3 function, the same phenomenon has recently been reported in other types of cancer. For example, Huang *et al* noted that 4T1 (a breast cancer cell line) and mouse embryonic fibroblast (MEF) cells showed a marked growth promoting ability towards the surrounding breast cancer cells when exposed to lethal doses of radiation (20). In addition, caspase 3 was demonstrated to be the key regulator in this tumor repopulation model by *in vitro* and *in vivo* evidence.

Furthermore, the presence of compensatory proliferation in dying ECs may aid in improving the understanding of therapy resistance in malignant glioma. One example is that the effect of anti-angiogenic agents, which mainly target vascular ECs during tumor therapy, may be compromised by growth stimulating signals released from dying ECs, according to the present findings. This may be one of the potential reasons why it is so difficult to obtain as high anti-angiogenic efficacy in clinical cases as is expected (21,22). Furthermore, there have also been several reports that certain tumors became more aggressive following treatment with angiogenic inhibitors in animal models, indicating the potential role of dying ECs during tumor progression (23,24). Thus, more attention should be paid to the compensatory signals from dying ECs and more *in vivo* experiments should be performed in the future to further study this mechanism.

Finally, the present study described a model of compensatory proliferation in tumors that was not only performed by damaged cancer cells or their supporting stromal cells, but also dying ECs. This method of compensatory proliferation was shown to be performed by the growth stimulating ability of ECs towards surrounding tumor cells via a caspase 3-mediated pathway. This phenomenon demonstrates the importance of blocking compensatory signals from dying cells during tumor therapy. Additionally, blocking caspase 3-mediated signaling pathways in combination with current tumor therapeutic strategy may be a promising approach for improving the dismal prognosis of malignant tumors.
